# Maternal Exposure to a Brominated Flame Retardant and Genitourinary Conditions in Male Offspring

**DOI:** 10.1289/ehp.0800058

**Published:** 2009-02-27

**Authors:** Chanley M. Small, John J. DeCaro, Metrecia L. Terrell, Celia Dominguez, Lorraine L. Cameron, Julie Wirth, Michele Marcus

**Affiliations:** 1 Department of Epidemiology; 2 Department of Human Genetics and; 3 Department of Gynecology and Obstetrics, Emory University, Atlanta, Georgia, USA; 4 Division of Environmental Health, Bureau of Epidemiology, Michigan Department of Community Health, Lansing, Michigan, USA; 5 Department of Epidemiology, Michigan State University, East Lansing, Michigan, USA

**Keywords:** brominated flame retardant, cryptorchidism, genitourinary condition, hernia, hydrocele, hypospadias, polybrominated biphenyls, urogenital abnormalities

## Abstract

**Background:**

The upward trend in industrial nations in the incidence of male genitourinary (GU) conditions may be attributed to increased exposure to endocrine disruptors. Polybrominated biphenyl (PBB), a brominated flame retardant, is one such suspected endocrine disruptor.

**Objective:**

We investigated the relationship between maternal serum levels of PBBs and GU conditions among male offspring exposed *in utero*.

**Methods:**

In this cohort study of sons born to women accidentally exposed to PBBs during 1973–1974, we examined self-reported data on GU conditions among male offspring in relation to maternal serum PBB levels. We used generalized estimating equations to calculate odds ratios (ORs), controlling for gestational age at birth.

**Results:**

Of 464 sons, 33 reported any GU condition (13 hernias, 10 hydroceles, 9 cryptorchidism, 5 hypospadias, and 1 varicocele). Four reported both hernia and hydrocele, and one both hernia and cryptorchidism. After adjustment for gestational age at birth, sons of highly exposed women (> 5 ppb) were twice as likely to report any GU condition compared with sons of the least exposed women [≤1 ppb; OR = 2.0; 95% confidence interval (CI), 0.8–5.1]. This risk was increased when we excluded sons born after the exposure but before the mother’s serum PBB measurement (OR = 3.1; 95% CI, 1.0–9.1). We found evidence of a 3-fold increase in reported hernia or hydrocele among sons with higher PBB exposure (test of trend *p*-value = 0.04). Neither hypospadias nor cryptorchidism was individually associated with PBB exposure.

**Conclusions:**

Although cryptorchidism and hypospadias were not associated with *in utero* PBB exposure, this study suggests that other GU conditions may be associated with exposure to endocrine-disrupting chemicals during development.

In the United States and in other industrialized nations, there appears to be an increase in the reported incidence of male genitourinary (GU) conditions such as cryptorchidism and hypospadias (Toppari et al. 2001). One hypothesis implicates *in utero* exposure to endocrine-disrupting chemicals, environmental contaminants that mimic or antagonize endogenous hormonal activity (Bay et al. 2006; Birnbaum and Cohen Hubal 2006; Sharpe 2006). Animal data strongly support the biological plausibility of this hypothesis (Bay et al. 2006; Sharpe 2006).

The Michigan Long-Term PBB Study provides an opportunity to prospectively assess the effects of human exposure to polybrominated biphenyl (PBB), a brominated flame retardant and suspected endocrine disruptor. In 1973–1974, Michigan residents were exposed to substantial PBB levels through contaminated animal and dairy products when NutriMaster, a nutritional cattle feed supplement, was inadvertently replaced with FireMaster, a brominated flame retardant (both from Michigan Chemical Corp., St. Louis, MI). Beginning in 1976, the Michigan Department of Community Health (MDCH) enrolled approximately 4,000 individuals with a range of exposure levels into a cohort study for long-term health monitoring (Fries 1985). This cohort of exposed individuals and their offspring (who were subsequently enrolled into the cohort) have been followed through the present time.

In the United States, PBB production ceased in 1979. However, concern remains for long-term health effects given continued production of structurally related organohalogen flame retardants [Agency for Toxic Substances and Disease Registry (ATSDR) 2004; Juhasz et al. 2007]. PBBs and similar organohalogens are stable, persistent pollutants possessing extremely long half-lives, with estimates ranging from 11 to 28 years, depending on the initial level of exposure (Blanck et al. 2000a; Rosen et al. 1995). Furthermore, PBBs are transferred across the placenta, and the fetus may be exposed *in utero* (Eyster et al. 1983; Jacobson et al. 1984). Prior investigations of *in utero* exposure to other organohalogens and the risk for GU conditions in boys are limited by case–control designs, with mixed results (Bhatia et al. 2005; Damgaard et al. 2006; Hosie et al. 2000; Longnecker et al. 2002). In a prospective study, Main et al. (2007) found that the content of polybrominated diphenyl ethers in maternal breast milk was higher among boys with cryptorchidism compared with boys without this condition. Similarly, Brucker-Davis (2008) found an association between cryptorchidism and polychlorinated biphenyls (PCBs).

The FireMaster mixture of PBBs may include congeners with estrogenic, anti-estrogenic, or antiandrogenic activity (McCormack et al. 1979; Nakari and Pessala 2005; Newton et al. 1982). Because of the possibility of varied effects, we began our study with a general exploratory hypothesis, that PBB exposure may affect a variety of GU conditions.

We previously found evidence of trans-generational endocrine effects of PBBs among female cohort members (Blanck et al. 2000b). We designed the present study to examine transgenerational endocrine effects among male cohort members. Specifically, we investigated *in utero* PBB exposure and reported GU conditions among the male offspring of cohort members.

## Materials and Methods

### Population

The institutional review boards at MDCH and at Emory University approved the study. The study population, drawn from the Michigan Long-Term PBB Study, included male offspring of female cohort members. Female cohort members were potentially exposed to PBB between the time when contaminated feed was delivered to Michigan farms (~ May 1973) and the time at which the farms were quarantined (~ May 1974). Because of the long half-life of PBB, these females maintained a body burden decades later and exposed subsequent pregnancies to PBB. Thus, we included in the study sons born after 1 July 1973 who had potential *in utero* PBB exposure. Participants gave either informed consent or assent.

### PBB exposure assessment

Maternal serum PBB levels were measured at the time in which the mother enrolled in the cohort using gas chromatography with electron- capture detection. PBB quantitation was based on PBB-153, the main congener involved in the PBB incident (Fries 1985). The limit of detection (LOD) for serum PBB was 1 ppb. Samples were collected from non-fasting participants, and serum lipid levels were not available.

We estimated a mother’s serum PBB level at the time of her son’s conception using a PBB decay model and used this estimation as a surrogate for her son’s *in utero* PBB exposure. Briefly, the decay model included body mass index (BMI) at initial PBB measurement, parity, age, smoking status, and breast-feeding. The decay model was validated using a subset of samples. The correlation between the predicted and observed concentrations was *r* = 0.93 (Terrell et al. 2008). The estimated date of conception was calculated by subtracting the gestational age from the date of birth. Although all mothers were exposed to PBB before the birth of their sons, not all mothers had their PBB measured before the birth of their sons. For most sons (*n* = 393), maternal PBB was measured before conception, permitting a prospective extrapolation of estimated maternal exposure at the time of the son’s conception. The estimated conception date for 71 sons, however, was after the exposure period start (1 July 1973) but before the mother’s blood was drawn for PBB measurement. In these cases, we used the PBB decay model to extrapolate backward to estimate maternal PBB at conception.

### Questionnaires

We assessed reporting of GU conditions among participants via a parent–son questionnaire sent to parents of sons 5–18 years of age and via telephone interview for adult sons (≥ 18 years). In the parent–son questionnaire, participants were asked specifically whether they had ever been told by a health care professional that they had varicocele/varicose veins in the scrotum, cryptorchidism/undescended testes, or hypospadias/abnormally placed urethral opening. In addition, the questionnaire included an open-ended question allowing individuals to report “any other conditions of the genitals or reproductive system.” Three age-specific versions contained minor wording differences. In the telephone interview, adult sons were asked whether they had ever been told by a health care professional that they had varicocele/varicose veins of the scrotum, undescended testes, or hypospadias. The telephone interview also included an open-ended question allowing adult sons to report “any other birth defect of the genital area.”

Questionnaires were sent to 375 sons 5–18 years of age with maternal PBB measurements, and 248 were completed (66%). Parents completed questionnaires for sons 5–10 years of age (*n* = 84). Parents and/or sons completed questionnaires for sons 11–16 years of age (*n* = 155). Sons 17 years of age (*n* = 9) completed questionnaires themselves.

Of the 363 adult sons we attempted to contact for telephone interview, 42 could not be located, 1 was deceased, and 75 with presumably correct contact information could not be reached. Of 245 contacted, 216 completed the telephone interview (88% contact response rate).

We obtained gestational age at the time of birth from birth certificates (66%). When birth certificates were not available, gestational age was determined from medical records (18%). If a medical record was not available, we obtained gestational age from a maternal telephone interview (15%). Similarly, birth weight was obtained from birth certificates (87%), a medical record (4%), or the PBB cohort infant enrollment form (8%).

### Statistical analysis

Data from parent–son questionnaires and telephone interviews were combined for analyses. Varicocele/varicose veins in the scrotum, cryptorchidism/undescended testes, and hypospadias/abnormally placed urethral opening were specifically addressed by the questionnaire and interview, and were included in the analyses. We included responses to “any other conditions (or birth defect) of the genitals or reproductive system” if determined to be a GU condition by physician reviewers (C.D. and J.J.D.).

We used both maternal enrollment PBB levels and estimated maternal levels at conception as surrogates for the son’s *in utero* exposure. PBB concentrations were categorized into three groups based on maternal enrollment levels: the LOD (≤ 1 ppb) and the median of detectable values among individuals reporting any GU condition (1–5 ppb, > 5 ppb). We calculated the frequency of each GU condition stratified by maternal PBB level.

We used generalized estimating equations (GEEs) to account for relatedness of brothers and adjusted for gestational age, and we calculated odds ratios (ORs) and 95% confidence intervals (CIs). We modeled the odds of developing any GU condition among all sons and compared these estimates with models excluding the 71 sons who were born after the exposure period but before their mothers’ PBB levels were measured. In addition, we modeled the odds of developing related GU conditions (cryptorchidism/hypospadias and hernia/hydrocele).

We examined confounding by gestational age, birth weight, and maternal age at conception. We did not assess confounding by race because 99% of the mothers in this cohort are Caucasian. We had limited information on smoking during pregnancy (only sons > 18 years of age were asked to respond to this question), so we were unable to assess confounding by maternal smoking during pregnancy. Calculations were performed using SAS, version 9.1 (SAS Institute Inc., Cary, NC).

## Results

Estimated maternal PBB at conception for younger sons who participated by parent–son questionnaire (mean = 10.9 ppb; range, ≤ LOD to 361 ppb) was lower than that of older sons who participated by telephone interview (mean = 13.1; range, ≤ LOD to 403 ppb); the median detectable maternal PBB level was 3.0 ppb among younger sons and 3.5 ppb among older sons.

The distribution of PBB concentrations did not differ between participants and nonparticipants. Tables 1 and 2 show characteristics of sons participating in the parent–son questionnaire and telephone interview, respectively. Neither gestational age (range, 34–45 weeks) nor birth weight (range, 964–5,868 g) differed by PBB level.

Of the 464 sons, 33 reported any GU condition, including 13 hernias, 10 hydroceles, 9 cases of cryptorchidism, 5 hypospadias, and 1 varicocele. Four individuals reported both hernia and hydrocele, and one reported both hernia and cryptorchidism. The incidences of GU conditions within the PBB long-term study were cryptorchidism (~ 19 in 1,000), hernia (~ 28 in 1,000), hydrocele (~ 22 in 1,000), and hypospadias (~ 11 in 1,000).

Table 3 shows the frequency of GU conditions stratified by maternal PBB level at enrollment and participant age; 7.7% of sons 5–18 years of age and 5.6% of sons 18–30 years of age reported any GU condition. Individuals with the highest maternal enrollment PBB levels (> 5 ppb) were more likely to report any GU condition than those with the lowest maternal levels (10.2% vs. 5.5%).

When we included all sons in the analysis, there was a nonsignificant 2-fold increase in the odds of GU conditions among sons in the highest PBB exposure group (for maternal enrollment PBB: OR = 2.0; 95% CI, 0.8–5.1; for estimated maternal PBB at conception: OR = 1.6; 95% CI, 0.6–4.3; Table 4). The effect of gestational age was in the expected direction. Sons born at ≤ 37 weeks of gestation were more likely to report a GU condition (OR = 2.6; 95% CI, 1.0–6.7). Adjusting for birth weight, gestational age, or maternal age at conception did not substantially alter the ORs for PBB exposure.

In models excluding the 71 sons who were born after the exposure period but before their mothers’ PBB was measured (Table 4), males with PBB exposure > 5 ppb were three times as likely to report any GU condition compared with those with the lowest exposure (≤ 1 ppb; for maternal enrollment PBB: OR = 3.1; 95% CI, 1.0–9.1; for estimated maternal PBB at conception: OR = 2.68; 95% CI, 0.9–7.9). Sons with a maternal enrollment PBB of 1–5 ppb were at slightly increased risk that did not reach statistical significance (for maternal enrollment PBB: OR = 2.1; 95% CI, 0.7–6.3; for estimated maternal PBB at conception; OR = 1.6; 95% CI, 0.6–4.4).

When grouped together, cryptorchidism and hypospadias did not show an association with PBB exposure (OR = 0.7; 95% CI 0.1–3.8 for the highest exposed compared with the least exposed). However, we found a 3-fold increase in reporting hydrocele or hernia among more highly exposed (*p*-value for trend = 0.04).

Of the individual conditions, only hernia appeared statistically associated with PBB exposure (Table 3). Among sons with the highest PBB exposure, 7.5% reported hernia compared with 1.4% in the lowest exposure group.

## Discussion

Our study suggests a possible association between reporting one or more GU conditions and *in utero* PBB exposure that should be replicated elsewhere. Boys born to mothers with high serum PBB levels were more than twice as likely to report any GU conditions. Interestingly, the association between PBB and hernia and hydrocele—conditions not often associated with endocrine disruption—drove this trend. To our knowledge, this is the first study demonstrating such an association for PBBs.

The overall incidence of GU conditions in the present study was comparable to expected rates in the general population. Specifically, cryptorchidism (~ 19 in 1,000) was comparable to estimates in the general population from cohort studies (~ 10–30 in 1,000) (Toppari et al. 2001). The incidence of hypospadias in the cohort (~ 11 in 1,000) is similar to current estimates that reflect an increase in the last few decades (2–8 in 1,000; Akre et al. 2008; Toppari et al. 2001). The overall incidence of hernia (~ 28 in 1,000) and of hydrocele (~ 22 in 1,000) was comparable to the estimated incidence for congenital hernia among U.S. live births (~ 10–20 in 1,000) (Davenport 1996).

We included all GU conditions reported by questionnaire or telephone interview regardless of *a priori* expectations regarding whether *in utero* PBB exposure would be a predisposing factor. Cryptorchidism and hypospadias, which result, respectively, from abnormalities in testicular descent (Hutson et al. 1997) and penile formation (Baskin and Ebbers 2006), are expected to be susceptible to *in utero* endocrine disruption. Hernia and hydrocele are also expected to be potentially susceptible to hormonal disruption related to failed testicular descent and cryptorchidism. Specifically, hernia and/or hydrocele occur when the processus vaginalis fails to close during normal testicular descent. This process is believed to be androgen dependent (Tanyel 2004). It is unexpected that we found no association between PBB exposure and either cryptorchidism or hypospadias.

We included varicocele despite a paucity of evidence regarding congenital risk factors. Inclusion of conditions neutral to the influence of endocrine disruptor exposures ought to bias toward the null. Interestingly, these conditions were reported only among sons in the highest exposure group. This may represent diagnostic bias among sons seeking care for other conditions. However, in the present study, varicocele was reported independently of other GU conditions, suggesting that endocrine disruptors may predispose to a wider variety of conditions than currently acknowledged.

Our finding of increased reports of hernia or hydrocele among the most exposed may be explained by an antiandrogenic effect of PBB. Animal data suggests antiandrogenic effects of perinatal PBB exposure through increased metabolism of testosterone and decreased responsiveness to testosterone (McCormack et al. 1979; Newton et al. 1982). The polychlorinated biphenyl (PCB) congener PCB-138 has demonstrated evidence of anti-androgenic activity (Bonefeld-Jorgensen et al. 2001), raising suspicion for antiandrogenic activity of PBB-138 (which accounts for 5–12% of the FireMaster mixture (Hass et al. 1978). It is plausible that PBBs exert indirect effects upon sex hormone activity through the inhibition or induction of CYP19 (aromatase) responsible for the conversion of androgens to estrogens [shown for the structurally similar compounds polybrominated diphenyl ethers by Canton et al. (2005)]. It is unlikely that the increase in hernia/hydrocele could be explained by the estrogenic activity of PBB-153 (Nakari and Pessala 2005) because we did not see an increased risk of cryptorchidism or hypospadias. Alternatively, the fact that we found increased incidence of hernia and hydrocele without an increase in cryptorchidism or hypospadias may indicate that the cause of this increase is through a route that is not hormonally mediated. PBBs and other organohalogens also interact with nonsex hormone receptors (e.g., the aryl hydrocarbon receptor) as well as induce cytochrome P450 (CYP) enzymes (ATSDR 2004). Thus, our findings might be explained by antiandrogenic activity of PBB-138 or other more complex mechanisms.

A major strength of the present study was its prospective design; women who were exposed to PBBs from 1973 to 1974 subsequently exposed sons *in utero*. Interestingly, the association between PBB and GU conditions was stronger among the 393 sons with whom we estimated PBB forward to the time of conception using a decay model. When the same model was used to backward extrapolate PBB at conception for an additional 71 sons, the effect of PBB on risk of GU conditions was attenuated. This difference either resulted from chance or may suggest backward extrapolation of estimated maternal PBB levels at conception is less accurate than forward predictions.

Furthermore, maternal PBB at enrollment showed a stronger relationship with GU conditions than estimated maternal PBB at conception. However, wide CIs indicate that there may be no real difference in these estimates, consistent with the long half-life of PBB. Like other studies, our assessment of *in utero* exposure was only indirect. Ethical and logistical limitations prevent more direct measurements.

Age at diagnosis was not available for many of the reported conditions. Cryptorchidism and hypo spadias, however, are always congenital. The descriptions of reported cases of hernia/hydrocele were most consistent with congenital forms of these disorders. Twelve individuals reporting a hernia/hydrocele included comments that indicated the conditions were diagnosed (or surgery was performed) before 5 years of age. However, one individual reported surgery at age 17. Of the remaining eight cases of hernia/hydrocele, all were interviewed between 5 and 15 years of age; therefore, the hernia/hydrocele would have been diagnosed before 15 years of age. The one case of varicocele reported an age at diagnosis of 17 years of age.

Nine individuals were born before April 1974 (9 months after July 1973) and may not have been exposed throughout their entire gestational period. None of these reported GU conditions. If these individuals biased our data, it would be toward the null if they did not have the opportunity for exposure. Because testicular descent occurs at the end of pregnancy, we did not want to exclude these individuals who were exposed during the later part of their gestation from our analyses.

Our study was limited by the use of self-reported data. The fewer cases of cryptorchidism reported by the 18- to 30-year-olds may be explained by the fact that these conditions were corrected (spontaneously or medically) during the first year of life and the older sons had no knowledge of this. In addition, cases of GU conditions may be underreported if only the more severe forms of the disorder were recalled. GU conditions may have been misclassified if, for example, a history of orchidopexy, which often includes ligation of a hernial sac, was recalled as a history of hernia and not as cryptorchidism. The effect of this mis classification should be minimal in our analysis of “any” reported GU condition. Among the 33 sons reporting any GU condition, 15 consented to a review of medical records. Of these, we received 13 records. Three contained insufficient information to confirm or refute the reported diagnosis. In the remaining 10 records, we confirmed the reported diagnoses. Finally, we cannot exclude reporting bias such that mothers and sons may be more inclined to remember and report a GU condition if they had a high PBB exposure. All participants in the PBB long-term study were informed of their serum PBB level shortly after enrollment.

Compared with prior studies, we were able to isolate the effects of a relatively homogeneous endocrine disruptor for which the precise exposure is known. However, we cannot exclude the possibility that multiple PBB congeners played a role or that other endocrine disruptors interacted with PBBs to result in the observed associations.

The increasing occurrence of male GU anomalies in industrialized countries warrants further study to guide public health efforts. In the present study, *in utero* PBB exposure was associated with reports of hernia and hydrocele. This suggests that endocrine disruptors may predispose to a wider variety of conditions than currently acknowledged.

## Figures and Tables

**Table 1:** Characteristics of males 5–18 years of age stratified by maternal serum PBB concentration^a^ at the time of enrollment into the cohort (*n* = 248).

		Maternal PBB concentration [*n* (%)]			
Characteristic	No. (%)	≤ LOD	1–3 ppb	>3 ppb	*p*-Value^b^
Age (years)					0.27

5–10	84 (33.9)	21 (30.4)	26 (28.9)	37 (41.6)	
11–13	67 (27.0)	16 (23.2)	26 (28.9)	25 (28.1)	
14–16	66 (26.6)	19 (27.5)	28 (31.1)	19 (21.4)	
17–18	31 (12.5)	13 (18.8)	10 (11.1)	8 (9.0)	

Gestational age (weeks)					0.15

34–37	29 (11.7)	9 (13.0)	8 (8.9)	12 (13.5)	
38–42	215 (86.7)	60 (87.0)	78 (86.7)	77 (86.5)	
43–45	4 (1.6)	0	4 (4.4)	0	

Birth weight (g)					0.56

964–2,499	5 (2.0)	1 (1.5)	2 (2.2)	2 (2.3)	
2,500–4,500	208 (83.9)	62 (89.9)	76 (84.4)	70 (78.7)	
4,501–5,216	10 (4.0)	2 (2.9)	2 (2.2)	6 (6.7)	
Missing	25 (10.1)	4 (5.8)	10 (11.1)	11 (12.4)	

a PBB categorized as ≤ LOD (1.0 ppb), > LOD to the median of detectable maternal values among participants (1–3 ppb), and above the median (> 3 ppb).

b Fisher’s exact *p*-value.

**Table 2:** Characteristics of males 18–31 years of age stratified by maternal PBB serum concentration^a^ at the time of enrollment into the cohort (*n* = 216).

		Maternal PBB concentration [*n* (%)]			
Characteristic	No. (%)	≤ LOD	1–3.5 ppb	> 3.5 ppb	*p*-Value
Age (years)					0.78^b^

18–19	30 (13.9)	11 (14.3)	8 (11.4)	11 (15.9)	
20–24	98 (45.4)	34 (44.2)	37 (52.9)	27 (39.1)	
25–29	74 (34.3)	27 (35.1)	22 (31.4)	25 (36.2)	
30–31	14 (6.5)	5 (6.5)	3 (4.3)	6 (8.7)	

Gestational age (weeks)					0.63

34–37	22 (10.2)	11 (14.3)	6 (8.6)	5 (7.3)	
38–42	173 (80.1)	59 (76.6)	56 (80.0)	58 (84.1)	
43–45	21 (9.7)	7 (9.1)	8 (11.4)	6 (8.7)	

Birth weight (g)					0.36^b^

1,928–2,499	7 (3.3)	1 (1.3)	1 (1.5)	5 (7.3)	
2,500–4,500	196 (91.2)	71 (92.2)	64 (92.8)	61 (88.4)	
4,501–5,868	12 (5.6)	5 (6.5)	4 (5.8)	3 (4.4)	

a PBB categorized as ≤ LOD of 1.0 ppb, > LOD to the median of detectable maternal values among participants (3.5 ppb), and above the median (> 3.5 ppb).

b Fisher’s exact *p*-value; chi-square *p*-value is the default.

**Table 3:** Frequencies [*n* (%)] of self-reported GU conditions in sons, stratified by age and maternal PBB serum concentration^a^ at the time of enrollment into the cohort (*n* = 464).

	All sons (5–30 years of age) (*n* = 464)				Sons 5–18 years of age (*n* = 248)				Sons 18–30 years of age (*n* = 216)			
	≤ LOD	1–5.0 ppb	> 5.0 ppb	*p*-Value	≤ LOD	1–5.0 ppb	> 5.0 ppb	*p*-Value	≤ LOD	1–5.0 ppb	> 5.0 ppb	*p*-Value
Any GU condition												

Yes	8 (5.5)	14 (6.6)	11 (10.2)	0.11^b^	4 (5.8)	12 (10.3)	5 (7.9)	0.55^b^	4 (5.2)	2 (2.1)	6 (13.6)	0.03
No	138 (94.5)	197 (93.4)	96 (89.7)		65 (94.2)	104 (89.7)	58 (92.1)		73 (94.8)	93 (97.9)	38 (86.4)	

Hernia												

Yes	2 (1.4)	3 (1.4)	8 (7.5)	0.01	0	3 (2.6)	3 (4.8)	0.14	2 (2.6)	0	5 (11.4)	0.002
No	144 (98.6)	208 (98.6)	99 (92.5)		69 (100)	113 (97.4)	60 (95.2)		75 (97.4)	95 (100)	39 (88.6)	

Hydrocele												

Yes	2 (1.4)	5 (2.4)	3 (2.8)	0.78	1 (1.5)	5 (4.3)	2 (3.2)	0.59	1 (1.3)	0	1 (2.3)	0.31
No	144 (98.6)	206 (97.6)	104 (97.2)		68 (98.5)	111 (95.7)	61 (96.8)		76 (98.7)	95 (100)	43 (97.7)	

Cryptorchidism												

Yes	3 (2.1)	5 (2.4)	1 (0.9)	0.83	3 (4.5)	3 (2.6)	1 (1.6)	0.70	0	2 (2.1)	0	0.69
No	141 (97.9)	204 (97.6)	105 (99.1)		64 (95.5)	111 (97.4)	61 (98.4)		77 (100)	93 (97.9)	44 (100)	

Hypospadias												

Yes	1 (0.7)	3 (1.4)	1 (0.9)	0.86	0	3 (2.6)	1 (1.6)	0.59	1 (1.3)	0	0	0.56
No	143 (99.3)	205 (98.6)	106 (99.1)		67 (100)	111 (97.4)	62 (98.4)		76 (98.7)	94 (100)	44 (100)	

Varicocele												

Yes	0	0	1 (0.9)	0.23	0	0	0		0	0	1 (2.3)	0.20
No	144 (100)	209 (100)	106 (99.1)		67 (100)	114 (100)	63 (100)		77 (100)	95 (100)	43 (97.7)	

a PBB categorized as ≤ LOD (1.0 ppb), > LOD to the median of maternal detectable PBB concentrations among cases (5 ppb), and greater than the median (> 5 ppb).

b Chi square *p*-value; Fisher’s exact *p*-values are the default.

**Table 4:** Adjusted ORs and 95% CIs for self-reported GU conditions.^a^

	Maternal enrollment PBB				Estimated maternal PBB at conception			
Condition	≤ LOD	1–5.0 ppb	> 5.0 ppb	Trend *p*-value	≤ LOD	1–5.0 ppb	> 5.0 ppb	Trend *p*-value
Any GU (*n* = 464)^b^	Reference	1.4 (0.5–3.5)	2.0 (0.78–5.1)	0.50	Reference	1.1 (0.4––2.5)	1.6 (0.6–4.3)	0.35
Any GU (*n* = 393)^c^	Reference	2.1 (0.7–6.3)	3.1 (1.0–9.1)	0.17	Reference	1.64 (0.6–4.4)	3.63 (0.9–7.9)	0.08
Cryptorchidism or hypospadias (*n* = 393)^c^	Reference	1.28 (0.4–4.7)	0.71 (0.1–3.8)	0.76	Reference	1.4 (0.4–4.8)	0.5 (0.1–4.7)	0.75
Hernia or hydrocele (*n* = 393)^c^	Reference	3.13 (0.4–23.9)	6.16 (0.9–42.5)	0.04	Reference	1.4 (0.3––6.0)	3.7 (0.9–16.0)	0.09

a Adjusted GEE models control for gestational age and the relatedness of siblings; the 5.0 ppb cut-point is the median level among the cases with detectable PBB.

b ***”***Any GU” includes hernia, hydrocele, cryptorchidism, hypospadias, and varicocele.

c Excluding 71 sons who were born after the exposure period but before the mothers’ initial serum PBB measurements.
